# Association between plasma trimethylamine N-oxide and cerebral white matter hyperintensity: a cross-sectional study

**DOI:** 10.3389/fnagi.2024.1498502

**Published:** 2024-12-04

**Authors:** Xiaotan Ji, Xudong Zhang, Jie Zhang, Shenna Niu, Hui Cong Xiao, Hong Chen, Chuanqiang Qu

**Affiliations:** ^1^Department of Neurology, Shandong Provincial Hospital, Shandong University, Jinan, China; ^2^Department of Neurology, Jining No. 1 People’s Hospital, Jining, China; ^3^Department of Neurology, Shandong Provincial Hospital Affiliated to Shandong First Medical University, Jinan, China; ^4^Department of Neurology, Zouping People’s Hospital, Binzhou, China; ^5^Clinical College of Neurology, Neurosurgery and Neurorehabilitation, Tianjin Medical University, Tianjin, China; ^6^Department of Emergency Center, Shandong Provincial Hospital Affiliated to Shandong First Medical University, Jinan, China

**Keywords:** trimethylamine N-oxide, TMAO, WMH, CSVD, MMP-9

## Abstract

**Background:**

Cerebral white matter hyperintensity (WMH) is a pivotal imaging feature of cerebral small vessel disease (CSVD), closely correlated with an elevated risk of ischemic stroke (IS). Trimethylamine N-oxide (TMAO), a metabolite of gut microbiota, is increasingly associated with IS and atherosclerosis. However, the intricate relationship between TMAO and WMH remains ambiguous. This study aimed to study the connection between plasma TMAO and WMH. Furthermore, it assessed the potential of TMAO as a risk evaluation instrument for WMH.

**Methods:**

In this cross-sectional study, we categorized WMH into periventricular WMH (P-WMH) and deep WMH (D-WMH), based on its locations. The severity of WMH was assessed and grouped according to the Fazekas scale. Plasma TMAO levels were quantitatively determined. We established the correlation between plasma TMAO levels and WMH severity using a Logistic regression model. Additionally, we employed ROC curves to evaluate the diagnostic efficacy of plasma TMAO concentration in distinguishing the severity of WMH.

**Results:**

A higher plasma TMAO tertile was significantly linked to a higher Fazekas score, encompassing the overall score, P-WMH score, and D-WMH score (*p* < 0.001). A logical regression analysis revealed that plasma TMAO levels were independently associated with overall moderate and severe WMH, compared to overall non-mild WMH, in the unadjusted model (OR = 1.373, 95%CI 1.183–1.594 for moderate; OR = 1.384, 95%CI 1.192–1.607 for severe), the adjusted model a (OR = 1.436, 95%CI 1.214–1.669 for moderate; OR = 1.446, 95%CI 1.222–1.711 for severe) and the adjusted model b (OR = 1.490, 95%CI 1.234–1.800 for moderate; OR = 1.494, 95%CI 1.237–1.805 for severe). The analysis also showed an independent correlation between plasma TMAO levels and WMH severity, irrespective of the unadjusted model, adjusted model a, or adjusted model b, when considering P-WMH and D-WMH severity. The ROC indicated that, in overall WMH and P-WMH, the area under curve (AUC) for non-mild and severe WMH were both>0.5, while the AUC for moderate WMH was<0.5. In contrast, in D-WMH, the AUC for non-mild, moderate, and severe WMH were all>0.5.

**Conclusion:**

Plasma TMAO levels exhibited a significant correlation with both overall and region-specific WMH severity. Furthermore, the plasma TMAO levels displayed robust predictive capability for D-WMH.

## Introduction

CSVD, a crucial contributor to IS and vascular dementia, encompasses the intricate interplay of diverse pathogenic factors that impact the entire cerebral vascular system, ranging from small arteries to venules. This intricate process leads to the manifestation of a myriad clinical presentations, radiographic abnormalities, and pathological states (Markus and de Leeuw, 2022). CSVD is associated with a variety of neuroimaging findings, including lacunar infarction, WMH, perivascular space enlargement, cerebral microbleeds, and cerebral atrophy (Cannistraro et al., 2019). Among them, WMH, colloquially known as cerebral white matter lesion (WML), stands as the most prevalent imaging manifestation of CSVD. This primarily denotes the presence of non-specific high signal intensities within the brain’s white matter (WM) tissue, as discernible on T2-weighted images or fluid-attenuated inversion recovery (FLAIR) magnetic resonance imaging sequences (Dadar et al., 2022). The prevalence of WMH intensifies with advancing age, particularly among the elderly population, where the incidence can soar up to an astonishing 90% (Huang et al., 2022). WMH is linked to a multitude of clinical manifestations, including gait and mood disorders, and stands as a prevalent cause of IS and dementia. Nevertheless, the incomplete understanding of the underlying mechanisms of WMH, coupled with the diverse array of influencing factors-both uncontrollable (e.g., age and gender) and controllable (such as diabetes and hypertension)-has led to a scarcity of effective therapeutic strategies to arrest or delay its progression (Han et al., 2022). Hence, it is imperative to delve into the pivotal risk factors and underlying mechanisms that drive the progression of WMH in individuals.

In recent years, there has been a growing body of research focused on the relationship between the gut microbial metabolite TMAO and neurological diseases. TMAO, a metabolite produced by the gut microbiota through the metabolism of nutrients like choline and carnitine commonly found in meat, fish, and eggs, has been shown to have a close association with the development of atherosclerosis and cardiovascular disease (CVD) (Vourakis et al., 2021; Zhen et al., 2023). Although previous research has demonstrated that TMAO holds a pivotal role in the inflammatory mechanisms underlying CVD and Alzheimer’s disease (AD) (Wang and Zhao, 2018; Arrona Cardoza et al., 2022), the connection between TMAO and WMH remains relatively understudied. The findings from a multi-center clinical study, encompassing 1,098 patients and conducted by Professor Wang Yilong’s team from Beijing Tiantan Hospital, revealed a significant correlation between elevated plasma levels of TMAO and an intensified signal of WMH in individuals diagnosed with CSVD (Chen et al., 2021). However, the precise connection between plasma TMAO levels and the severity of WMH remains elusive. Consequently, this study endeavors to delve into the correlation between plasma TMAO levels and WMH severity, while also engaging in a deeper discussion of the potential mechanisms of TMAO in WMH pathogenesis.

## Materials and methods

### Research design

This cross-sectional study encompassed 112 WMH patients. All patients fulfilled the ethical standards set by the Ethics Committee of Shandong Provincial Hospital Affiliated to Shandong First Medical University. Furthermore, all participants voluntarily signed the Informed Consent Form. The subjects comprised adult patients admitted to the Department of Neurology, Shi Zhong District People’s Hospital, Jinan, Shandong Province, between January and December 2023. Upon admission, fundamental subject data was gathered, encompassing name, age, gender, body mass index (BMI), blood pressure (BP), and a comprehensive history of prior health conditions, such as hypertension (HP), diabetes mellitus (DM), coronary heart disease (CHD), hyperlipidemia (HLP), and glomerular filtration rate (eGFR). Additionally, the smoking and drinking patterns of the patients were meticulously evaluated. The inclusion criteria stringently stipulated an age threshold of 18 years, confirmation of WMH via FLAIR, and consistent Fazekas scale scores. Conversely, exclusion criteria excluded individuals with severe organ insufficiency, alternative forms of cognitive decline, and a history of severe cardiovascular or cerebrovascular disorders. From eligible patients, blood plasma samples were collected early in the morning after admission, and subsequently stored in a −80°C refrigerator for further analysis.

### The MR T2 FLAIR sequence

All participants were scanned using a 3.0 Tesla brain MR scanner (Philips, Achieva) with a 32-channel head coil, encompassing axial T1-weighted imaging (T1WI), axial T2-weighted imaging (T2WI), and FLAIR sequence imaging with a slice thickness of 3 mm. The FLAIR imaging is a 3D sequence with multiplanar reformations. All MR scanners and Fazekas score assessment were done by 2–3 neuroradiologists, ensuring the evaluators were blinded to the participants’ basic information. The diagnostic criteria for WMH serve as a reference and involve the identification of scattered dot-like, block-like, or large-scale high-signal intensities located around or within the bilateral regions of the brain, based on intracranial MRI examination findings.

### WMH severity grading

The categorization of WMH has been previously detailed in other clinical trials (Yu et al., 2021), our study employed comparable assessment techniques to categorize overall Fazekas scores into three groups: non-mild WMH (Fazekas score 0–2), moderate WMH (Fazekas score 3–4), and severe WMH (Fazekas score 5–6). Based on the severity of P-WMH and D-WMH, they were further classified as non-mild (Fazekas score 0–1), moderate (Fazekas score 2), and severe (Fazekas score 3). The Fazekas scores of P-WMH and D-WMH were summed up to derive an overall Fazekas score, ranging from 0 to 6.

### TMAO and MMP-9 determination

The concentrations of plasma TMAO and MMP-9 were quantitatively determined utilizing the Enzyme-linked immunosorbent assay (ELISA) technique. To summarize, the process entailed pre-coating a specific antibody, subsequently adding the sample and horseradish peroxidase (HRP), with 3,3′, 5,5′-tetramethylbenzidine (TMB) serving as the substrate. The absorbance (OD value) was then measured at 450 nm, utilizing a standard curve for comparison.

### Statistical analysis

Categorical variables were represented using proportions, while continuous variables with a normal distribution were characterized by the mean ± standard deviation. For continuous variables with a non-normal distribution, the median (interquartile range) was utilized. The statistical evaluations were executed utilizing SPSS 25.0, GraphPad Prism 10, and R Studio. Comparisons among continuous variables were facilitated by the ANOVA, Kruskal-Wallis test, or Mann–Whitney U test, as applicable. Categorical variables were scrutinized using the Pearson Chi-square test. Furthermore, three logistic regression models were devised to gauge the risk of WMH in relation to TMAO. The basic model entailed no adjustments, model a adjusted for gender and pivotal cardiovascular risk factors (HP, DM, CHD, HLP), and model b further considered BMI > 24 kg/m^2^ and eGFR<90 mL/min. In the regression analysis, the OR value and its 95% CI were computed. The Spearman rank correlation was employed to ascertain the link between plasma TMAO levels and Fazekas scores. The receiver operating characteristic (ROC) curves for non-mild, moderate, and severe WMH, P-WMH, and D-WMH were generated using R Studio software. The area under the ROC curve (AUC) was calculated to forecast the severity of WMH by evaluating plasma TMAO levels. All tests were conducted as two-sided, and statistical significance was deemed at *p* < 0.05.

## Results

### Baseline characteristics of the study cohort

A total of 112 patients were enrolled in our study, with a male predominance of 77.0% and a median age of 65 years. During the period spanning from December 2023 to January 2024, comprehensive tests were conducted, encompassing plasma MMP-9 levels, BMI, BP measurements, and detailed blood analyses. The latter encompassed various blood components such as white blood cells, red blood cells, hemoglobin, platelets, and lymphocytes, as well as assessments of renal function (specifically, blood urea nitrogen and eGFR), lipid profiles (including cholesterol, triglycerides, HDL, LDL, homocysteine), glycemic markers (fasting blood glucose, glycated hemoglobin), and thyroid function indicators (free triiodothyronine, free thyroxine, and thyroid stimulating hormone).

The mean concentrations of plasma MMP-9 and TMAO among the participants were 20.88 ng/mL and 25.03 ppm, respectively. For TMAO levels, the cohort was stratified into three quartiles: a low group with TMAO concentrations ≤21.56 ppm (*n* = 38), a middle group with concentrations ranging from 21.56 ppm to 25.16 ppm (*n* = 37), and a high group with concentrations ≥25.16 ppm (*n* = 37). Notably, no statistically significant differences were observed among these groups in terms of gender distribution, HP status, DM prevalence, or the presence of CVD.

### The association between plasma TMAO levels and the severity of overall WMH

**Figure 1:**
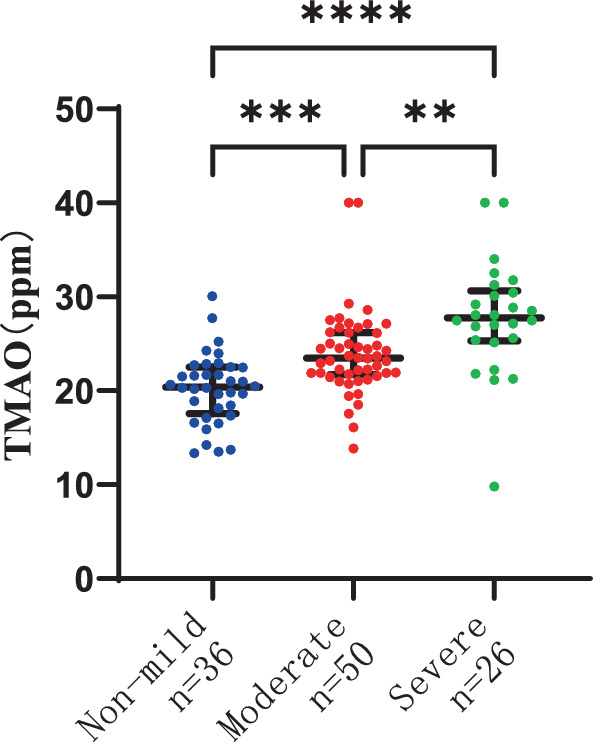
Differences in plasma TMAO levels among groups of overall white matter hyperintensity.

There were 36 patients with non-mild overall WMH, 50 patients with moderate overall WMH, and 26 patients with severe overall WMH. The severity of overall WMH was notably correlated with plasma TMAO levels in patients, with statistical significance at *p* < 0.001. Furthermore, the severity of overall WMH was linked to elevated plasma MMP-9 levels and age, with a significance level of *p* < 0.05. The plasma TMAO levels were significantly higher in patients with moderate WMH compared to those with non-mild WMH, as indicated by the median values of 23.48 (IQR 21.71–25.25) ppm versus 20.41 (IQR 17.55–22.51) ppm, respectively, at *p* < 0.001. Similarly, patients with severe WMH exhibited higher plasma TMAO levels than those with non-mild WMH, with median values of 27.7 (IQR 25.33–30.66) ppm versus 20.41 (IQR 17.55–22.51) ppm, respectively, at *p* < 0.001. Additionally, patients with severe WMH had significantly higher plasma TMAO levels compared to those with moderate WMH, with median values of 27.78 (IQR 25.33–30.66) ppm versus 23.48 (IQR 21.71–25.25) ppm, respectively, at *p* < 0.001 (Figure 1).

**Figure 2:**
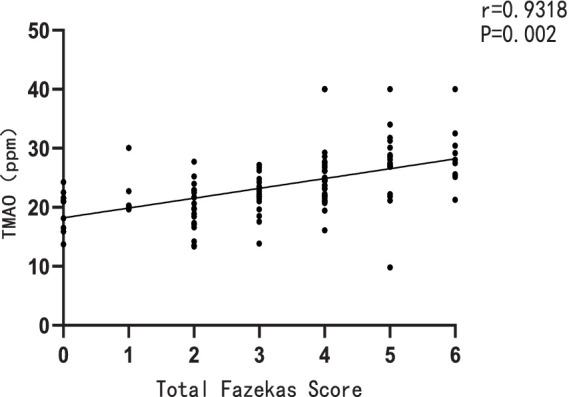
Correlation between plasma TMAO levels and total Fazekas score.

**Figure 3:**
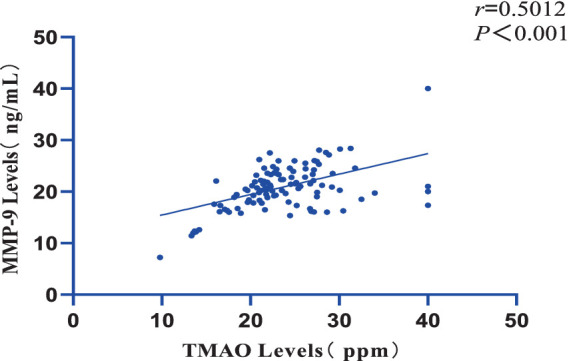
Correlation between plasma TMAO levels and MMP-9 levels.

Furthermore, a significant positive correlation was observed between the plasma TMAO levels and the overall Fazekas score, with a correlation coefficient of r = 0.9318 and a significance level of *p* = 0.002 (Figure 2). There was also a notable positive correlation between plasma TMAO levels and MMP-9 levels, with a correlation coefficient of *r* = 0.5012 and a significance level of *p* < 0.001 (Figure 3).

**Figure 4:**
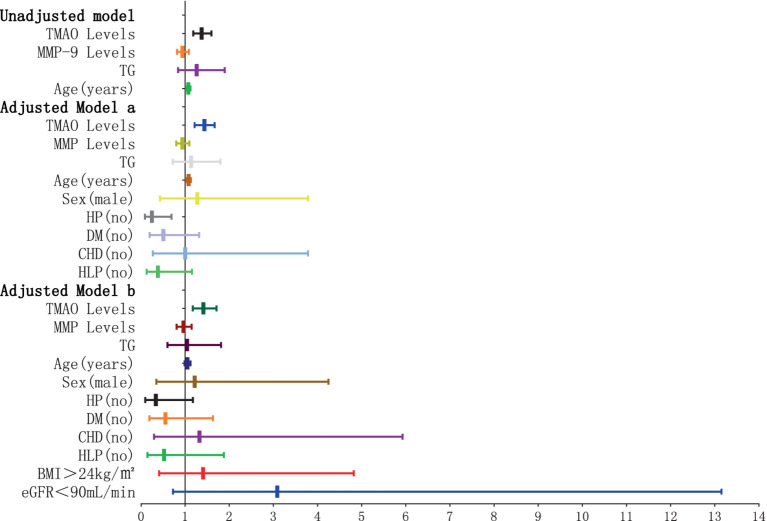
Forest plot of the association between plasma TMAO levels and overall moderate white matter hyperintensity. *x*-axis: 95% CI; y: regression model, including unadjusted model: TMAO levels, MMP-9 levels, TG (no) and Age (years). Adjusted model a: TMAO levels, MMP-9 levels, TG (no), Age (years), Sex (male), and cerebrovascular risk factors (HP, DM, CHD, HLP) (no). Adjusted model b: TMAO levels, MMP-9 levels, TG (no), Age (years), Sex (male), cerebrovascular risk factors (HP, DM, CHD, HLP) (no), BMI > 24 kg/m^2^, and eGFR<90 mL/min. When the 95% CI on the *x*-axis includes 1, and the horizontal line segment in the forest plot intersects the null line, it can be considered that the difference has no statistically significant; when the 95% CI on the *x*-axis is <1, and the horizontal line segment in the forest plot does not intersect the null line and is to the left of the null line, it can be considered that the factor is a protective factor for overall moderate WMH. When the 95% CI on the *x*-axis is >1, and the horizontal line segment in the forest plot does not intersect the null line and is to the right of the null line, it can be considered that the factor is a risk factor for overall moderate WMH.

**Figure 5:**
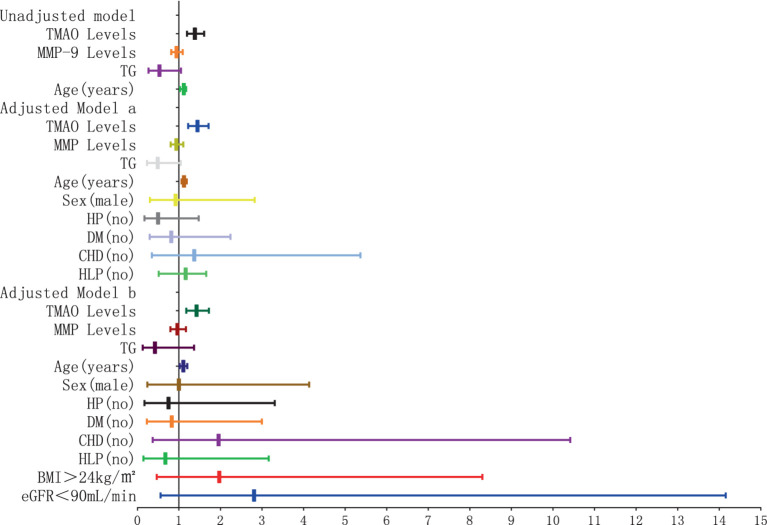
Forest plot of the association between plasma TMAO levels and overall severe white matter hyperintensity.

The findings of the logistic regression analysis, examining the link between plasma TMAO levels and the severity of overall WMH, are presented in Table 1 and Figures 4, 5. Essentially, in the unadjusted model (comprising plasma TMAO levels, plasma MMP-9 levels, TG, and age), a marked association was observed between plasma TMAO levels and moderate to severe WMH, as opposed to non-mild WMH (moderate WMH: OR = 1.373, 95% CI: 1.183–1.594; severe WMH: OR = 1.384, 95% CI: 1.192–1.607). Similarly, age independently correlated with WMH severity (moderate WMH: OR = 1.072, 95% CI: 1.026–1.120; severe WMH: OR = 1.116, 95% CI: 1.062–1.173).

**Table 1:** Logistic regression analysis of the association between plasma TMAO levels and the severity of overall white matter hyperintensity.

	Moderate			Severe		
	*p*-value	OR value	95%CI	*p*-value	OR value	95%CI
Unadjusted Model						
TMAO	<0.001	1.373	1.183–1.594	<0.001	1.384	1.192–1.607
MMP-9	0.393	0.940	0.817–1.083	0.418	0.942	0.816–1.088
TG	0.265	1.261	0.839–1.895	0.069	0.531	0.268–1.050
Age (years)	0.002	1.072	1.026–1.120	<0.001	1.116	1.062–1.173
Adjusted Model a						
TMAO	<0.001	1.436	1.214–1.669	<0.001	1.446	1.222–1.711
MMP-9	0.415	0.937	0.800–1.096	0.451	0.940	0.800–1.104
TG (no)	0.591	1.134	0.716–1.797	0.063	0.490	0.231–1.040
Age (years)	0.004	1.075	1.023–1.129	<0.001	1.123	1.063–1.187
sex (male)	0.662	1.275	0.429–3.785	0.884	0.920	0.300–2.825
HP (no)	0.008	0.245	0.087–0.692	0.208	0.499	0.169–1.473
DM (no)	0.162	0.506	0.194–1.316	0.690	0.814	0.296–2.241
CHD (no)	0.999	1.001	0.265–3.784	0.654	1.368	0.348–5.370
HLP (no)	0.088	0.384	0.128–1.155	0.266	0.515	1.160–1.658
Adjusted Model b						
TMAO	<0.001	1.415	1.171–1.711	<0.001	1.422	1.175–1.721
MMP-9	0.670	0.962	0.807–1.148	0.708	0.964	0.794–1.170
TG	0.883	1.043	0.599–1.815	0.149	0.421	0.130–1.363
Age (years)	0.130	1.050	0.986–1.119	0.014	1.106	1.020–1.199
sex (male)	0.760	1.216	0.347–4.246	0.997	0.998	0.241–4.133
HP (no)	0.088	0.336	0.096–1.178	0.704	0.750	0.170–3.303
DM (no)	0.284	0.555	0.189–1.630	0.773	0.827	0.229–2.993
CHD (no)	0.713	1.325	0.296–5.927	0.433	1.953	0.366–10.418
HLP (no)	0.320	0.522	0.145–1.878	0.616	0.673	0.143–3.162
BMI > 24 kg/㎡	0.589	1.405	0.409–4.823	0.355	1.972	0.468–8.300
eGFR<90 mL/min	0.127	3.088	0.725–13.155	0.211	2.807	0.556–14.158

BMI > 24 kg/m^2^: Overweight; eGFR < 90 mL/min: Low Glomerular Filtration Rate. Unadjusted Model: TMAO concentration, MMP-9 concentration, TG (no), and Age (years); Adjusted Model a: TMAO concentration, MMP-9 concentration, TG (no), Age (years), Sex (male), and cerebrovascular risk factors (HP, DM, CHD, HLP); Adjusted Model b: TMAO concentration, MMP-9 concentration, TG (no), Age (years), Sex (male), cerebrovascular risk factors (HP, DM, CHD, HLP), BMI > 24 kg/m^2^, and eGFR < 90 mL/min. When *p*-value < 0.05, the difference is statistically significant; OR > 1 indicates a risk factor, OR < 1 indicates a protective factor. When *p*-value > 0.05, the difference is not statistically significant. 95% CI represents the confidence interval.

In the adjusted model a, where gender and cerebrovascular risk factors (HP, DM, CHD, HLP) were taken into account, plasma TMAO levels retained its significant association with WMH severity (moderate WMH: OR = 1.436, 95% CI: 1.214–1.669; severe WMH: OR = 1.446, 95% CI: 1.222–1.711), and the correlation with age persisted (moderate WMH: OR = 1.075, 95% CI: 1.023–1.129; severe WMH: OR = 1.123, 95% CI: 1.063–1.187).

Furthermore, in model b, which additionally considered BMI > 24 kg/m^2^ and eGFR<90 mL/min, the independent relationship between plasma TMAO levels and moderate to severe WMH remained statistically significant (moderate WMH: OR = 1.415, 95% CI: 1.171–1.711; severe WMH: OR = 1.422, 95% CI: 1.175–1.721). However, the association of age with severe WMH in this model was noted with a minor adjustment in the CI range (OR = 1.016, 95% CI: 1.020–1.199).

### The association between plasma TMAO levels and the severity of P-WMH and D-WMH

To delve deeper into the correlation between plasma TMAO levels and the severity of WMH in distinct regions, we segregated the study participants into two groups: P-WMH and D-WMH. Specifically, the P-WMH group comprised 30 non-mild, 56 moderate, and 26 severe subjects. Notably, individuals with moderate and severe P-WMH exhibited a greater tendency to have elevated levels of age and SP in comparison to those with non-mild P-WMH, with statistical significance (*p* < 0.05). Shifting focus to the D-WMH classification, we identified 56 non-mild, 46 moderate, and 10 severe subjects. Our analysis revealed a marked association between plasma MMP-9 levels and D-WMH severity, again with statistical significance (*p* < 0.05). Furthermore, patients with moderate and severe D-WMH were significantly more likely to be of advanced age compared to those with non-mild D-WMH, achieving a higher level of statistical significance (*p* < 0.005).

**Figure 6:**
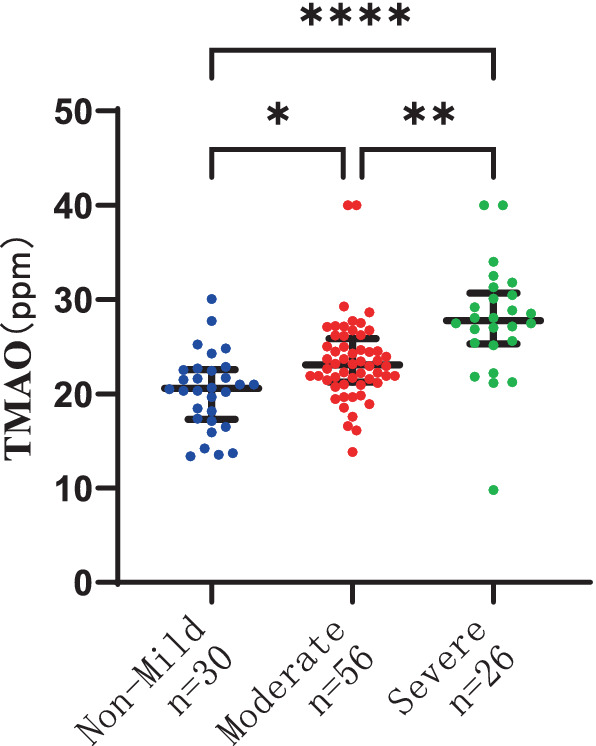
Differences in plasma TMAO levels among groups in periventricular white matter hyperintensity.

**Figure 7:**
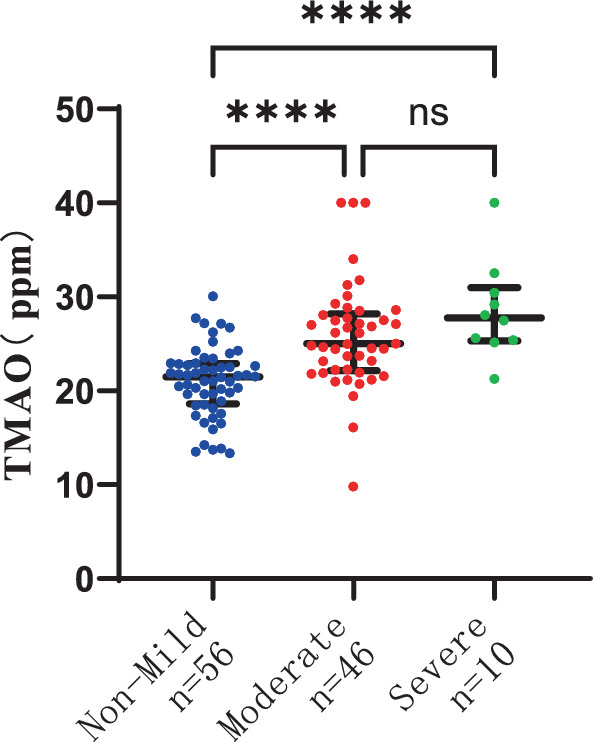
Differences in plasma TMAO concentration among groups in deep white matter hyperintensity.

The severity of WMH, both P-WMH and D-WMH, positively correlates with the levels of TMAO in plasma. Specifically, the plasma TMAO levels were significantly higher in patients with moderate P-WMH compared to those in the non-mild P-WMH group [median 23.04 (IQR 21.23–25.86) ppm versus median 20.57 (IQR 17.29–22.58) ppm, *p* < 0.05]. Furthermore, patients with severe P-WMH exhibited even higher plasma TMAO levels than those with non-mild P-WMH [median 27.78 (IQR 25.33–30.66) ppm versus median 20.57 (IQR 17.29–22.58) ppm, *p* < 0.0001], and the same trend was observed when comparing severe P-WMH patients to those with moderate P-WMH [median 27.78 (IQR 25.33–30.66) ppm versus median 23.04 (IQR 21.23–25.86) ppm, *p* < 0.01] (Figure 6). Similarly, the plasma TMAO levels were significantly elevated in patients with moderate D-WMH compared to the non-mild D-WMH group [median 25.00 (IQR 22.13–28.19) ppm versus median 21.46 (IQR 18.62–22.91) ppm, *p* < 0.0001]. This trend continued in patients with severe D-WMH, who showed even higher plasma TMAO levels than those with non-mild D-WMH [median 27.78 (IQR 25.33–30.97) ppm versus median 21.46 (IQR 18.62–22.91) ppm, *p* < 0.0001]. However, no statistically significant correlation was found between the plasma TMAO levels in patients with severe D-WMH and those with moderate WMH (Figure 7).

**Figure 8:**
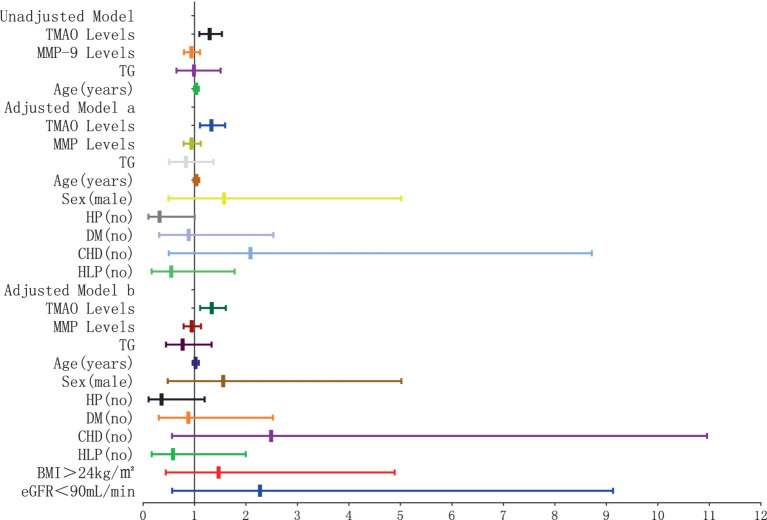
Forest plot of the association between plasma TMAO levels and moderate periventricular white matter hyperintensity.

**Figure 9:**
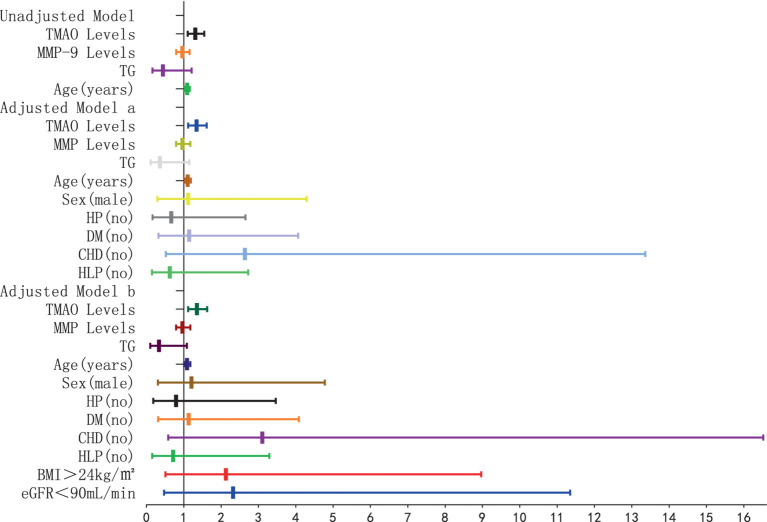
Forest plot of the association between plasma TMAO levels and severe periventricular white matter hyperintensity.

The results of the logistic regression analysis examining the relationship between plasma TMAO levels and the severity of P-WMH are depicted in Figures 8, 9. In the unadjusted model, plasma TMAO levels were significantly and independently associated with both moderate (OR = 1.295, 95% CI: 1.095–1.531) and severe P-WMH (OR = 1.307, 95% CI: 1.104–1.547). Furthermore, age was exclusively and independently associated with severe P-WMH (OR = 1.092, 95% CI: 1.024–1.164). Upon adjustment in model a, taking into account sex (male) and cerebrovascular risk factors including HP, DM, CHD, and HLP, plasma TMAO levels retained their independent association with moderate (OR = 1.330, 95% CI: 1.107–1.538) and severe P-WMH (OR = 1.343, 95% CI: 1.118–1.614). Age also remained independently associated with severe P-WMH (OR = 1.106, 95% CI: 1.030–1.188). In the second adjusted model b, similar findings were observed, with plasma TMAO levels independently associated with moderate (OR = 1.336, 95% CI: 1.109–1.609) and severe P-WMH (OR = 1.349, 95% CI: 1.119–1.626) in patients. Age, once again, was independently associated with severe P-WMH in this group (OR = 1.088, 95% CI: 1.006–1.177).

**Figure 10:**
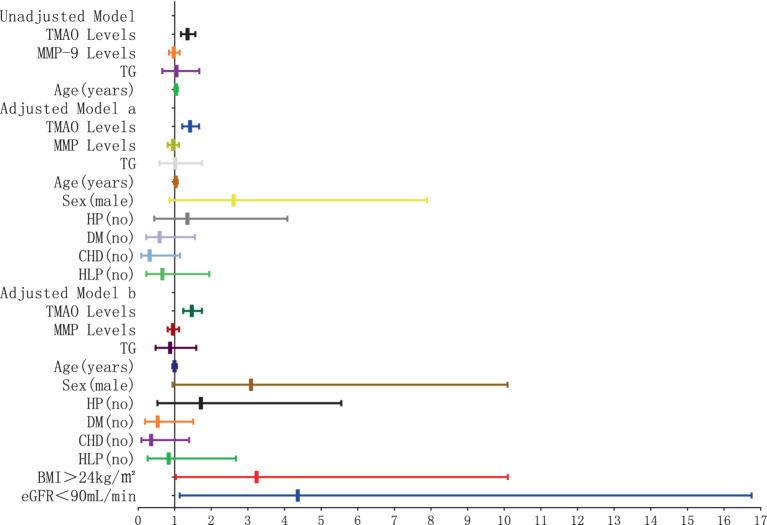
Forest plot of the association between plasma TMAO levels and moderate deep white matter hyperintensity.

**Figure 11:**
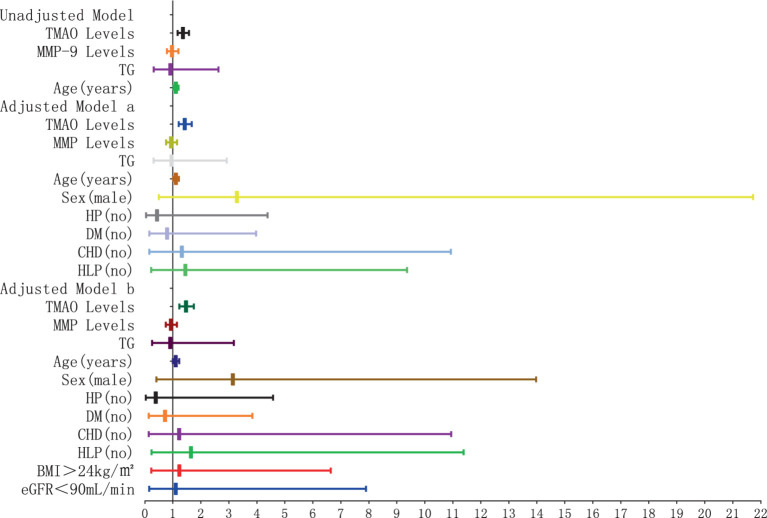
Forest plot of the association between plasma TMAO levels and severe deep white matter hyperintensity.

The results of the logistic regression analysis examining the relationship between plasma TMAO levels and the severity of D-WMH are depicted in Figures 10, 11. In the unadjusted model, it was found that plasma TMAO levels were independently correlated with moderate D-WMH (OR = 1.352, 95%CI 1.170–1.562) and severe D-WMH (OR = 1.357, 95%CI 1.170–1.573). Additionally, age emerged as an independent predictor for severe D-WMH (OR = 1.108, 95%CI 1.019–1.204). Upon adjustment in model a, which accounted for gender (male) and cerebrovascular risk factors (HP, DM, CHD, HLP), plasma TMAO levels remained independently associated with moderate D-WMH (OR = 1.419, 95%CI 1.209–1.665) and severe D-WMH (OR = 1.419, 95%CI 1.207–1.672). Age also continued to be independently associated with severe D-WMH (OR = 1.108, 95%CI 1.010–1.216). In the adjusted model b, which further controlled for BMI > 24 kg/m^2^and eGFR<90 mL/min, plasma TMAO levels were independently associated with both moderate D-WMH (OR = 1.468, 95%CI 1.235–1.744) and severe D-WMH (OR = 1.469, 95%CI 1.233–1.751). Furthermore, BMI > 24 kg/m^2^ and eGFR<90 mL/min were found to be independently associated with moderate D-WMH (BMI: OR = 3.236, 95%CI 1.037–10.096; eGFR: OR = 4.360, 95%CI 1.135–16.752).

### Analysis of the receiver operating characteristic curve for WMH severity and plasma TMAO levels

**Figure 12:**
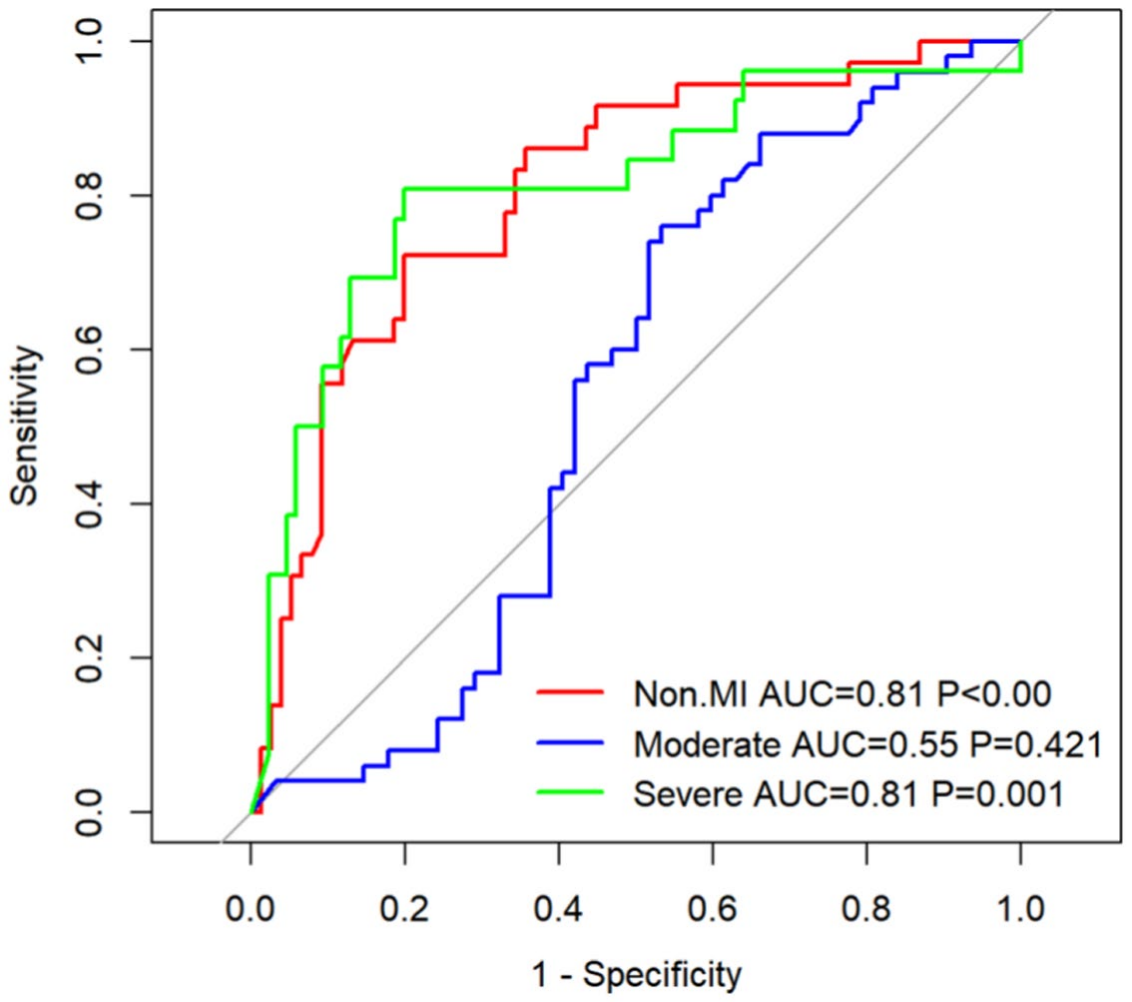
(A) ROC curve of the overall white matter hyperintensity subjects. (B) ROC curve of the paraventricular white matter hyperintensity subjects. (C) ROC curve of the deep white matter hyperintensity subjects.

The ROC curve was employed to assess the diagnostic efficacy of plasma TMAO levels in discerning the severity of WMH. The ROC analysis for overall WMH revealed an AUC of 0.81 for both non-mild and severe WMH, with a significance level of *p* < 0.05. Simultaneously, the AUC for moderate cases was 0.55, indicating a lack of statistical significance at *p* = 0.421 (Figure 12A). For P-WMH, the ROC curve yielded an AUC of 0.77 for non-mild and 0.81 for severe WMH, both with *p* < 0.05. The AUC for moderate cases was 0.55, which was not statistically significant at *p* = 0.421 (Figure 12B). In the case of D-WMH, the ROC curve demonstrated AUC values of 0.81, 0.72, and 0.72 for non-mild, moderate, and severe subjects, respectively, all with *p* < 0.001 (Figure 12C).

## Discussion

Our cross-sectional study employed a targeted metabolomics analysis to investigate the correlation between plasma TMAO levels and the severity of WMH. The findings indicated a positive correlation between these factors. Even after accounting for gender, cerebrovascular risk factors such as HP, DM, CHD, HLP, BMI, and eGFR, plasma TMAO levels maintained an independent association with moderate to severe WMH. Notably, plasma TMAO levels exhibited robust predictive capabilities in patients with D-WMH, and in those with P-WMH, it was particularly relevant for patients with moderate to severe lesions. Furthermore, plasma TMAO levels displayed a significant positive correlation with MMP-9 levels. In summary, plasma TMAO levels are significant in evaluating the severity of WMH in patients and lay the groundwork for further exploration into the potential mechanisms by which TMAO influences WMH.

The severity of WMH frequently serves as an indirect measure for evaluating the integrity of normal brain white matter and as a surrogate biomarker for CSVD (Maniega et al., 2015; Maltais et al., 2019), involved in the pathological processes of cognitive decline and dementia (Inaba et al., 2023). WMH primarily manifests as demyelination and axonal loss, which are consequences of chronic cerebral ischemia. We have previously carried out pertinent clinical and fundamental research. Upon analyzing the clinical case data, the findings indicated that patients with hypertensive WMH exhibited elevated plasma TMAO levels compared to those with hypertensive non-WMH and healthy volunteers without hypertension. In our fundamental experiments, it was observed that Spontaneously Hypertensive Rats (SHRs) at 35 weeks of age presented with severe WMH, accompanied by higher TMAO levels in both blood and cerebrospinal fluid, as opposed to SHRs at 24 weeks (Ji et al., 2022). A recent meta-analysis has indicated that TMAO serves as a risk factor for stroke, demonstrating a positive correlation between elevated plasma TMAO levels and an increased risk of stroke, particularly in the case of arteriole occlusion subtype (Farhangi et al., 2020; Xu et al., 2022). A recent cross-sectional study revealed that the plasma levels of TMAO in patients diagnosed with acute ischemic stroke were associated with lacunar stroke, yet no such association was observed with other subtypes of ischemic stroke, including cardiogenic stroke. In the multivariate analysis, the research further indicated a correlation between elevated plasma TMAO levels and an increased volume of high signal intensity in WMH (Kijpaisalratana et al., 2023), which is consistent with the viewpoint of our study. Related studies suggest that TMAO may accelerate the atherosclerotic process and is associated with an increased risk of cardiovascular disease (Andò et al., 2014; Wang et al., 2014; Koeth et al., 2018). However, an experimental study in a mouse model showed that gut dysbiosis may further worsen WMH by exacerbating neuroinflammation during chronic insufficient cerebral blood flow, which underscores the importance of the gut-brain axis in the regulation of CSVD (Inaba et al., 2023). Based on this, we speculated that TMAO in the gut-brain axis correlated with WMH severity, which neuroinflammation may play a key role in the development of WMH.

TMAO is a metabolite produced by dietary meat, fish, and eggs, including nutrients such as choline and carnitine. These compounds are first converted into trimethylamine (TMA), rapidly absorbed into the blood, and converted into TMAO in the liver through the flavin monooxygenase system (FMO) (Zeisel and Warrier, 2017; Wang et al., 2019). In addition, TMA may also be degraded to methylamine, dimethylamine (DMA), and ammonia in the colon (Subramaniam and Fletcher, 2017; Gessner et al., 2020; Papandreou et al., 2020). The substance was subsequently eliminated via body fluids and feces. A diet rich in choline and carnitine contributed to the production of TMA and TMAO. An increase in TMAO concentration may be caused by diet, changes in gut microbiota composition, gut dysbiosis, or damage to the gut-blood barrier. Recent studies utilizing a mouse model have demonstrated that the gut microbial CutC gene can influence the production of TMA and TMAO, which may subsequently impact stroke severity and result in unfavorable functional outcomes (Zhu et al., 2021). Notably, this is consistent with several recent clinical studies showing an association between elevated plasma TMAO levels and an increased risk of stroke and adverse clinical outcomes in subjects with a transient ischemic attack (TIA) or IS (Rexidamu et al., 2019; Zhai et al., 2019; Schneider et al., 2020). Dietary supplementation with TMAO, carnitine, or choline can reduce reverse cholesterol transport *in vivo* and promote vascular atherosclerosis, which is associated with the occurrence of cardiovascular and cerebrovascular diseases (Koeth et al., 2013). Epidemiological studies have shown that both a Western diet and red meat-rich diets can significantly increase plasma TMAO levels and have a significant association with stroke risk (Wolk, 2016; Schwingshackl et al., 2019; Jain et al., 2020). Several observational studies also showed a correlation between the cerebral small vessel imaging marker WMH and plasma TMAO levels (Chen et al., 2021; Luciani et al., 2023), which is in agreement with our study results. Therefore, dietary intervention in patients with a high risk of stroke may help to delay the development of WMH, which is our next research direction.

WMH is a common imaging feature of CSVD, and its development is influenced by multiple risk factors, among which hypertension is one of the key factors. However, hypertension is often caused by atherosclerosis, and its effective management can not only alleviate the aggravation of WMH, but also reduce the risk of angiogenic cognitive impairment (van Middelaar et al., 2018). Nevertheless, the current treatment for WMH is still limited and the pathology of WMH is poorly understood. Associated with the pathogenesis of WMH, high plasma levels of TMAO led to increased permeability of BBB and inflammation in the central nervous system (Dolkar et al., 2024). Our previous study showed that TMAO was able to induce NLRP 3 inflammasome activation by promoting increased mitochondrial reactive oxygen species (Ji et al., 2022). This study focused on TMAO, to explore its association with WMH and to assess whether TMAO could be used as a predictor of WMH severity. Below, we will discuss the underlying pathogenesis between TMAO and WMH from the following two aspects.

On the one hand, the BBB, as a pivotal defense mechanism of the central nervous system, is comprised of endothelial cells within the brain. It is constructed in concert with astrocytes and other neurons to safeguard the brain against pathogens and noxious substances (Obermeier et al., 2013). The integrity of BBB is crucial for preserving the stability of the brain’s environment. Recent studies have indicated that enhancing endothelial function can mitigate white matter damage in rat models of CSVD. These findings suggested that endothelial cell dysfunction might precede the onset of CSVD, potentially even before the disruption of the BBB (Rajani et al., 2018). Moreover, the earliest known pathophysiological alteration in WMH is BBB dysfunction (Debette and Markus, 2010). Recent research indicates that physiologically relevant levels of TMAO can bolster the integrity of BBB and shield it from inflammatory damage. Conversely, elevated concentrations of TMAO compromise BBB function and disrupt the integrity of tight junctions (Hoyles et al., 2021). However, related studies showed that TMA is harmful to the BBB, and TMAO itself is protective to the BBB, which improved BBB integrity *in vitro* and *in vivo* and promoted cognitive performance in mice (Ahmed et al., 2022). Whether TMAO leads to neuroinflammation and aggravates WMH by destroying BBB, the specific molecular mechanism needs further study.

On the other hand, neuroinflammation plays a key role in the development of WMH and cognitive dysfunction. As the primary immune cells in the brain, astrocytes and microglia are the major drivers of early neuroinflammation. Our previous *in vivo* and *in vitro* studies showed that TMAO under hypertensive conditions can significantly increase reactive oxygen species in cells and activate the NLRP3 inflammasome, thereby impairing mitochondrial function and causing inflammatory death in oligodendrocytes, subsequently exacerbating the severity of WMH (Ji et al., 2022). This suggested that TMAO is able to promote cerebral white matter demyelination, but the specific inflammatory response pathway still needs to be further defined. When the integrity of BBB is impaired, neurotoxic molecules, blood cells, including immune cells, and various pathogens can enter the brain and trigger an inflammatory response (Inaba et al., 2023). Moreover, TMAO also exacerbated the release of inflammatory mediators by inhibiting the expression of FTO and IGF2BP2 and promoting the activation of NLRP3 inflammasome, thus promoting further damage and increasing the permeability of BBB (Ge et al., 2023). Neuroinflammation may result in the prolonged activation of microglia and the emergence of pathological conditions, particularly within WMH. Conversely, activated microglia can reciprocally affect the BBB, potentially causing its dysfunction (Candelario-Jalil et al., 2022). For example, microglia can release the MMP-9, which destroys the extracellular matrix of the vascular endothelium, promotes the destruction of the BBB, increases permeability, and intensifies WMH (Cannistraro et al., 2019). This has similarities with our study, where high plasma levels of MMP-9 showed a significant positive correlation with WMH severity.

### Limits

Our study has deepened our understanding of the relationship between TMAO and WMH; however, our analysis suggests several areas that warrant further exploration. Initially, although an association between TMAO and WMH has been established, the development and progression of WMH is a complex, multifactorial process that involves BBB dysfunction, neuroinflammatory responses, and other mechanisms that are not yet fully understood. Future studies should adopt a longitudinal design to further investigate whether there is a causal relationship between TMAO and the progression of WMH, and to explore the specific molecular pathways involved. Secondly, the sample size of this study was relatively small and did not include TMAO analysis in cerebrospinal fluid and feces, which limits a comprehensive understanding of TMAO’s role in WMH. Considering CSF samples can directly reflect the biochemical environment within the brain, and fecal samples can provide insights into gut microbial composition, the examination of these specimens may be crucial in understanding the biological effects of TMAO. Additionally, there may be significant inter-individual variations in gut microbial composition and hepatic metabolic capacity, which could introduce greater variability in outcomes when TMA or TMAO is measured independently. The TMA/TMAO ratio might be a better predictor of disease risk development compared to TMAO alone. Pursuing these research directions will be essential in identifying potential intervention targets for WMH and in revealing the mechanisms by which TMAO contributes to the onset and progression of WMH.

## Data Availability

The original contributions presented in the study are included in the article/supplementary material, further inquiries can be directed to the corresponding author.
